# GRK5 – A Functional Bridge Between Cardiovascular and Neurodegenerative Disorders

**DOI:** 10.3389/fphar.2018.01484

**Published:** 2018-12-17

**Authors:** Jhana O. Hendrickx, Jaana van Gastel, Hanne Leysen, Paula Santos-Otte, Richard T. Premont, Bronwen Martin, Stuart Maudsley

**Affiliations:** ^1^Department of Biomedical Science, University of Antwerp, Antwerp, Belgium; ^2^Center for Molecular Neurology, University of Antwerp – Flanders Institute for Biotechnology (VIB), Antwerp, Belgium; ^3^Institute of Biophysics, Humboldt-Universitat zu Berlin, Berlin, Germany; ^4^Harrington Discovery Institute, Case Western Reserve University, Cleveland, GA, United States; ^5^Faculty of Pharmaceutical, Biomedical and Veterinary Sciences, University of Antwerp, Antwerp, Belgium

**Keywords:** G-protein coupled receptor kinase 5, aging, cardiovascular disease, neurodegeneration, GRK5 interactors

## Abstract

Complex aging-triggered disorders are multifactorial programs that comprise a myriad of alterations in interconnected protein networks over a broad range of tissues. It is evident that rather than being randomly organized events, pathophysiologies that possess a strong aging component such as cardiovascular diseases (hypertensions, atherosclerosis, and vascular stiffening) and neurodegenerative conditions (dementia, Alzheimer’s disease, mild cognitive impairment, Parkinson’s disease), in essence represent a subtly modified version of the intricate molecular programs already in place for normal aging. To control such multidimensional activities there are layers of trophic protein control across these networks mediated by so-called “keystone” proteins. We propose that these “keystones” coordinate and interconnect multiple signaling pathways to control whole somatic activities such as aging-related disease etiology. Given its ability to control multiple receptor sensitivities and its broad protein-protein interactomic nature, we propose that G protein coupled receptor kinase 5 (GRK5) represents one of these key network controllers. Considerable data has emerged, suggesting that GRK5 acts as a bridging factor, allowing signaling regulation in pathophysiological settings to control the connectivity between both the cardiovascular and neurophysiological complications of aging.

## Introduction

### GPCR Signaling

Heptahelical G protein-coupled receptors (GPCRs) represent one of the largest superfamilies of transmembrane receptor proteins. Rough estimates suggest the presence of between 800 and 900 somatic (i.e., outside of odorant receptors; Fredriksson et al., 2003; Matthews and Sunde, 2012) GPCR genes, or just over 1% of protein-generating genome. These plasma membrane GPCRs facilitate cell sensitivity to a diverse array of external stimuli including light, small chemical transmitters and large glycoprotein hormones (Takeda et al., 2002). GPCRs undergo a conformational change in response to an impinging regulator, allowing them to serve as guanyl nucleotide exchange factors for heterotrimeric GTP-binding proteins (G-proteins) resulting in their dissociation (Cabrera-Vera et al., 2003). Dissociated heterotrimeric α and βγ subunits can then productively interact with downstream effectors to generate soluble second messenger molecules (e.g., calcium or inositol trisphosphate) to engender a broad range of biological actions (Blank et al., 1992; Exton, 1996; Maudsley et al., 2004; Hewavitharana and Wedegaertner, 2012). In addition to their capacity to confer sensitivity to stimuli involved in productive cellular signaling, GPCR systems also form a crucial part of stress response pathways linked to disease-propagating pathophysiologies.

### G Protein-Coupled Receptor Kinases Modulate GPCR Signaling

GPCR-regulated heterotrimeric G protein signaling termination, as a specifically organized molecular event, has been described by many research teams and is now considered canon for most GPCRs (Zhang et al., 1999). After the initial ligand induced conformational change of the receptor and G protein complex, there is a recruitment of a class of serine/threonine kinases to the active receptor, i.e., G protein-coupled receptor kinases (GRKs). This association is initiated by interaction between the free G protein βγ subunits and a pleckstrin homology (PH) domain present within the GRK protein. Upon association, the GRK typically phosphorylates the receptor on available serine or threonine residues within an acidic amino acid context (Aspartate or Glutamate) found in the receptors three intracellular loops or the carboxyl-terminus (C-terminus). This phosphorylation both attenuates subsequent association with GDP-bound Gαβγ G protein heterotrimers, while promoting the association of β-arrestin molecules with the activated and phosphorylated receptor. This stable β-arrestin association serves to further inhibit subsequent G protein heterotrimer association and simultaneously increase the ability of the receptor to interact with components of endocytic systems such as clathrin and the AP-2 adaptor protein (Laporte et al., 1999). Therefore, via both GRK phosphorylation and subsequent β-arrestin association, the ligand-induced receptor activity is considered to be quenched and eventually terminated – this process has been classically referred to as agonist-induced receptor desensitization (Maudsley et al., 1998; Zhang et al., 1999). The desensitization process is vital to maintain continued physiological responsivity to stimuli and to protect the responding cell/tissue against protracted deleterious exposure to ligands. GRKs are not the only kinase type that can phosphorylate GPCRs after ligand activation, so-called heterologous (as opposed to GRK homologous) receptor phosphorylation can be mediated by second messenger-dependent protein kinases, e.g., protein kinase A or C (Steele et al., 2002). Subsequent research, however, has demonstrated that this generic desensitization process is not simply a signal termination event, as more of a signal modulation event. Hence, this canonical GRK-β-arrestin cascade actually transmutes the G protein signaling identity of the receptor to a non-G protein signaling mode, involving GRKs, β-arrestins and other GPCR-associated signaling molecules (Hall et al., 1998; Luttrell et al., 1999; Cant and Pitcher, 2005; van Gastel et al., 2018b). With specific respect to the interactions between GRK and β-arrestin signaling dynamics, there are further levels of nuance in this paradigm. Hence, it has been shown that GPCRs can be regulated by a variety of interacting GRKs, where some display a relative selectivity of interaction, while others demonstrate a promiscuity of association. In this scenario, differential phosphorylation patterns upon the receptor loops or C-terminus can engender a specific downstream effect on subsequent β-arrestin-based signaling cascades (Nobles et al., 2011; Lefkowitz, 2013).

Due to their ability to control a wide array of biological functions, GPCR-based signaling activity has become perhaps the primary target for effective therapeutic research. GPCR-based regulation of cellular pathological mechanisms has shown tremendous clinical relevance for neurodegenerative (Maudsley et al., 2007; Huang et al., 2017), metabolic (Martin et al., 2017; Riddy et al., 2018), neoplastic (Liu et al., 2016), respiratory (Douthwaite et al., 2017), and cardiovascular (Conrad, 2016) disorders. As studies continue to uncover further nuances in GPCR regulatory behavior, new roles, aside from simple receptor phosphorylation, for GRKs in GPCR systems have emerged. It is now apparent from many excellent studies that GRKs also control intermediary cellular metabolic signaling pathways, independent of GPCR functionality. It is in this regard that our review will focus, especially with respect to the body’s capacity to control complex multi-tissue processes – such as aging – through coordination via systemic “keystone” proteins that possess a trophic level of control over coherent signaling networks.

### Keystone Control of Cardiovascular Disease and Neurodegeneration Communication

At the present time there is considerable evidence emerging that suggests a functional connectivity between blood vessel damage due to arterial stiffness (Cefalu, 2011; Vlachopoulos et al., 2015; Costantino et al., 2016) and resultant dementia (van Sloten et al., 2015; Cooper and Mitchell, 2016; Pase et al., 2016; de Roos et al., 2017). Multiple molecular mechanisms that interconnect these two pathophysiological domains have been proposed based on the evidence of both *in cellulo* and *in vivo* animal studies. Arterial stiffness is a condition linked to the age-dependent pathological elevation of pulse wave velocity (PWV). Both murine and human studies have suggested that increases of PWV can lead to an increased incidence of cognitive decline and dementia (O’rourke and Hashimoto, 2007; Avolio, 2013; Marfella and Paolisso, 2016; Ryu et al., 2017). In addition to elevated PWV, arterial stiffness can result in a potentiated pulsatile flow in a broad range of cerebral blood vessels. Increased blood flow pulsatility has been shown to engender the creation of cellular reactive oxygen species (ROS) that, upon interaction with vascular and neuronal proteins, can induce cerebral gliosis and endothelial dysfunction with concomitant dysregulated blood brain-barrier (BBB) permeability. These deleterious effects of ROS can often cause a functional deficit within cerebral perfusion circuits, that both attenuate the distribution of metabolic fuels, while simultaneously reducing the ability to clear neurotoxic chemical products – these two events then conspire to promote cognitive dysfunction and dementia (Sadekova et al., 2013; Iulita et al., 2017). It is widely accepted that complex age-related disorders represent highly interconnected molecular events spanning multiple tissues. We have demonstrated previously that in order to control these processes “keystone” proteins, that coordinate self-reinforced signaling networks in these processes, can be identified and potentially targeted therapeutically to attempt to mitigate these systems-level disorders (Lu et al., 2015; Martin et al., 2016; van Gastel et al., 2018a).

Aging, as a pathological process represents perhaps the strongest risk factor for both cardiovascular disease (CVDs) and neurodegenerative conditions (Kennedy et al., 2014). Indicative of the strong etiological role of the pathological aging process in the generation of cardiovascular diseases it has been shown that many of the well-characterized “hallmarks of aging” (López-Otín et al., 2013) are prominent in multiple CVD paradigms. Hence CVDs are often strongly associated with telomere attrition (De Meyer et al., 2018), epigenetic alterations (Rando and Chang, 2012), proteostasis alterations (Wiersma et al., 2016), disrupted nutrient sensing (Uryga and Bennett, 2016), mitochondrial dysfunction (Marzetti et al., 2013), and cellular senescence (Donato et al., 2018). Such a profound intersection is also observed between classical neurodegenerative pathways and those typically associated with pathophysiological aging. For example, aging has been correlated with the occurrence of several types of dementia, affecting 5–10% of people over 65, and about 50% of people over 85 years old (Prince et al., 2015). Alzheimer’s disease (AD), perhaps one of the most common forms of dementia, shares multiple functional overlaps with canonical brain aging pathways including mitochondrial dysfunction (Müller et al., 2018), oxidative stress (Watts et al., 2018), calcium management alterations (Popugaeva et al., 2018) and impaired proteostasis (Chadwick et al., 2012; Martínez et al., 2017). Given the significant molecular connections between aging pathomechanisms and these two prevalent disease realms (CVD and neurodegeneration), the likelihood that there are physical bridges between these signaling domains is high. In this review, we will particularly discuss an incipient role of GRK5 in the context of a potential role for this GPCR-associated signaling factor as a trophic coordinator of both cardiovascular and neurodegenerative pathophysiologies.

### G-Protein Coupled Receptor Kinases

GRKs are found nearly universally in complex organisms ranging from non-metazoans to vertebrate mammals (Mushegian et al., 2012). Currently there are known to be seven different GRK types, i.e., GRK1 to GRK7 (Kohout and Lefkowitz, 2003; Penela et al., 2003). Across these different types there is a shared 60–70% sequence homology. The seven GRK members are divided into three subfamilies based on their sequence homology (Premont and Gainetdinov, 2007). The rhodopsin kinase or visual GRK subfamily comprises GRK1 and GRK7. The β-adrenergic receptor kinases subfamily, comprising GRK2 and GRK3 have been most widely studied due to their strong association with canonical agonist-induced desensitization pathways. Finally, the GRK4 subfamily, comprises the kinases GRK4, 5, and 6. Here in this review we will focus upon the multidimensional functionalities of one of these isoforms, i.e., GRK5. With respect to their somatic distribution, near ubiquitous somatic expression has been shown for GRKs 2, 3, 5, and 6. The expression of GRK1 and GRK7 is restricted the retina (Hisatomi et al., 1998), while GRK4 expression is predominantly found in testicular, renal and cerebellar tissues (Premont et al., 1996; Sallese et al., 1997).

As previously discussed, the primary role conceptualized for GRKs was their ability to actively phosphorylate-signaling cell surface GPCRs (Pitcher et al., 1998; Penela et al., 2003; Lefkowitz and Shenoy, 2005). This GRK-mediated phosphorylation was then considered to simply attract β-arrestins to the active state receptor to further inhibit G protein-mediated signaling, through the so-called process of homologous receptor “desensitization.” As a result of this desensitization process, phosphorylated receptors can then be targeted for endocytic removal from the plasma membrane via clathrin- or caveolae mediated processes. The fate of the internalized receptor is then sensitive to the degree of prevailing ligand stimulation – moderate cell surface receptor stimulation allows for recycling of the receptor to the surface for re-engagement with a ligand, while protracted excessive ligand stimulation overloads the recycling machinery and results in GPCR targeting for lysosomal degradation (Pitcher et al., 1998). While agonist-induced desensitization was considered to represent a signaling termination event, groundbreaking research demonstrated that indeed components of the desensitizing molecular machinery actual constitute a further mode of signaling of the “desensitized” receptor (Maudsley et al., 1998; Luttrell et al., 1999). Thus, pro-desensitizing β-arrestin activity represents only a small component of its activity and instead arrestin signaling itself seems to be potentially independent of G protein signaling and facilitates the creation of ligand-induced GPCR-based adaptor protein scaffolds. As well as possessing a potent role in the regulation of GPCR signaling dynamics, both GRKs and β-arrestins are also important signal conditioning factors in receptor tyrosine kinase signaling cascades (Hildreth et al., 2004; Robinson and Pitcher, 2013; Zhang et al., 2015). Therefore, it is clear that our appreciation of GRK functionality may need to include diverse cellular activities and also subcellular localities. For example, while the majority GRKs are predominantly cytosolic or found in proximity to the plasma membrane, GRK5 is also often concentrated in the cellular nucleus (Martini et al., 2008; Gold et al., 2013). Along with this re-appraisal of canonical GRK functionality, it was originally proposed that the GRK only phosphorylates the active ligand-bound GPCR (Li et al., 2015). With particular respect to this, it has been shown that members of the GRK4 subfamily (including GRK5) can also effectively phosphorylate inactive GPCR structures. This ligand-independent GRK phosphorylation still retains some of the aspects of the canonical agonist-induced desensitization paradigm, i.e., subsequent β-arrestin recruitment to the inactive yet GRK-phosphorylated receptor is still evident.

In addition to these non-canonical GRK functions, these multifunctional kinases can also control the ability of GPCRs to demonstrate biased signaling, dependent on the GRK-specific phosphorylation patterns generated in the receptor primary sequence (Choi et al., 2018). These phosphoprotein patterns can effectively determine which β-arrestin isoforms (β-arrestin1 or 2) are recruited, and which specific super-structure conformations these eventual receptor-arrestin complexes then adopt (Lee et al., 2016). In this context therefore, the GRK interaction with receptors has a profound effect upon subsequent downstream non-G protein-dependent signaling cascades emanating from GPCRs. Therefore, both the qualitative and quantitative aspects of GPCR signaling (at both the G protein-dependent and –independent level) are likely to be a function of the relative expression level, catalytic activity, interactomic associations and subcellular localization of GRKs. In the following sections, the GRK5 protein itself, numerous GRK5 interacting proteins and GRK5’s biological role in the context of connecting multiple age-related disease paradigms will be discussed.

### Molecular Functionality of GRK5

**FIGURE 1:**
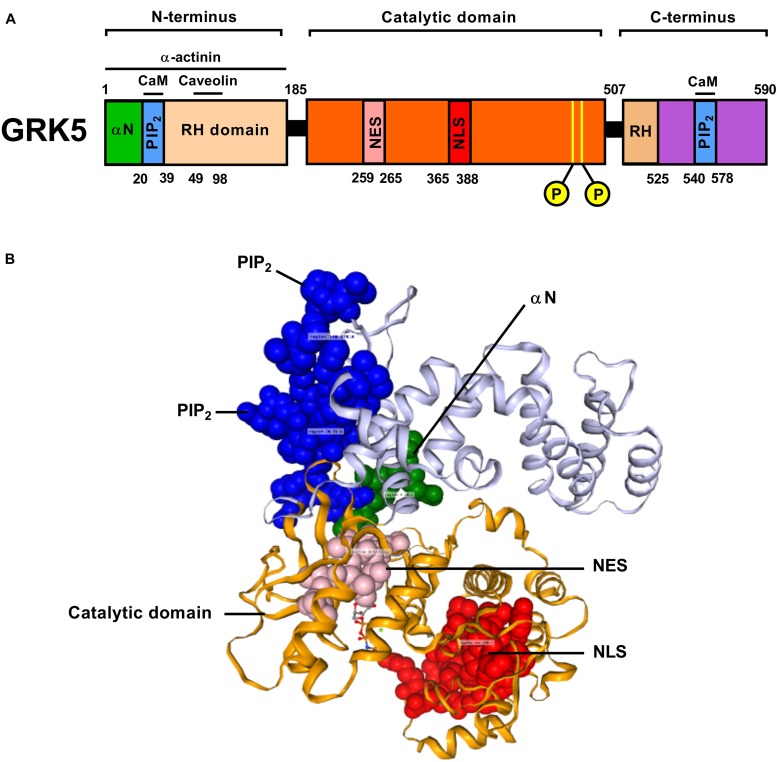
A graphical block depiction **(A)** of the multiple functional regions of human GRK5 indicates the diverse range of activities mediated by this protein (α-N, terminal domain; PIP2, phosphatidylinositol bisphosphate binding domain; RH, Regulator of G protein signaling homology domain; NES, Nuclear Export Sequence; NLS, Nuclear Localization Sequence). In the primary sequence two autophosphorylation sites are indicated within the catalytic domain. A crystal-assisted model of human GRK5 **(B)** was constructed and annotated using 3DBionotes-WS (http://3dbionotes.cnb.csic.es/ws). This model indicates the three-dimensional proximities of the α-N domain (green), PIP2 binding domains (blue), NES (pink), catalytic domain (orange), and the NLS domain (red).

GRK5 is composed of 500–700 amino acids and shares multiple common features with other members of the GRK superfamily (Figure 1). Thus, GRK5 possesses a central catalytic domain (∼270 residues), surrounded by a C-terminal domain of variable length (∼105–230 residues). GRK5, like the other members of the GRK4 subfamily possesses a specific amino-terminal (N-terminal) domain (∼185 residues). GRK5 possesses an amphipathic helix membrane binding domain, located in its C-terminal RH (RGS homology) domain, which is important for its function and proper localization at the plasma membrane (Pitcher et al., 1992; Koch et al., 1993; Premont et al., 1999; Kohout and Lefkowitz, 2003; Thiyagarajan et al., 2004; Penela et al., 2006; Xu et al., 2014). In contrast, the N-terminal domain of GRK5 appears to be important for intracellular membrane localization as well as for receptor recognition (Murga et al., 1996). The N-terminus also contains an RH domain (∼120 residues) (Kohout and Lefkowitz, 2003; Penela et al., 2003) as well as a phosphatidylinositol (4,5) bisphosphate (PIP2) region that can influence the kinases catalytic activity (Pitcher et al., 1998). Recently, GRK5 has been crystalized in two unique monomeric structures with consistent C-terminal structures closely packed to the RH domain. Individual subunits of the GRK5 architecture have been shown to be insufficient for persistent membrane association since disruption of the C-terminus/RH domain interface significantly decreases the GRK5 catalytic activity on GPCRs (Xu et al., 2014). Both the C- and N-terminal motifs predominantly localize GRK5 to the plasma membrane (at the expense of cytosolic concentration), which in turn facilitates its ability to control activation-independent phosphorylation activity at GPCRs (Li et al., 2015). GRK5 is subtly different from the other GRK4 subfamily members due to its possession of a nuclear localization sequence (NLS) motif. This NLS motif enhances the ability of GRK5 to translocate to the nucleus where it can exert non-canonical GRK activities, e.g., it has been shown that nuclear GRK5 can act as a histone deacetylase kinase and thus control gene transcription activity (Johnson et al., 2004; Martini et al., 2008). Binding of Ca^2+^ and calmodulin (CaM) to GRK5 has been shown to inhibit GRK5’s membrane association, thus augmenting its nuclear localization (Gold et al., 2013). Hence it is apparent that the nuclear translocation of GRK5 likely exists in competition with its membrane localization. For example, C-terminal protein kinase C (PKC)-mediated phosphorylation attenuates its nuclear functional activity.

In addition to their classical roles in GPCR signaling cascades, work in the past decade has shown that GRKs also productively interact with many signaling factors outside this paradigm (Kurose, 2011; Gurevich et al., 2012; Hullmann et al., 2016). A broad range of GRK5 binding partners have been identified via different molecular biological approaches, e.g., Affinity Purification Mass Spectrometry and yeast-two hybrid screens. With the advent of well curated interactomic metadata for signaling proteins it is now relatively simple to appreciate that for many signaling systems, e.g., the GRK pathway, the extent of protein–protein interactions can significantly expand the potential of such multidimensional molecules to control both health and disease in a manner outside of their canonical activities. GRK5 interacting partners currently include single transmembrane receptors (Freedman et al., 2002; Hildreth et al., 2004) as well as cytosolic (Liu et al., 2005; Barthet et al., 2009; Lafarga et al., 2012) and nuclear proteins (Parameswaran et al., 2006; Martini et al., 2008). The productive interaction of GRKs with intracellular non-GPCR proteins profoundly influences diverse transduction pathways (Ho et al., 2005; Peregrin et al., 2006; Sorriento et al., 2008; Barthet et al., 2009; Wang et al., 2009; Patial et al., 2010) including cell cycle (Penela et al., 2010; Michal et al., 2012), apoptosis (Chen et al., 2010), cell motility (Penela et al., 2008; Lafarga et al., 2012), and inflammation (Sorriento et al., 2008; Patial et al., 2010). The study therefore of the functional GRK5 interactome, will likely help elucidate both novel mechanisms of intrinsic protein regulation as well as to further clarify GRK5-associated physiological signaling properties.

**FIGURE 2:**
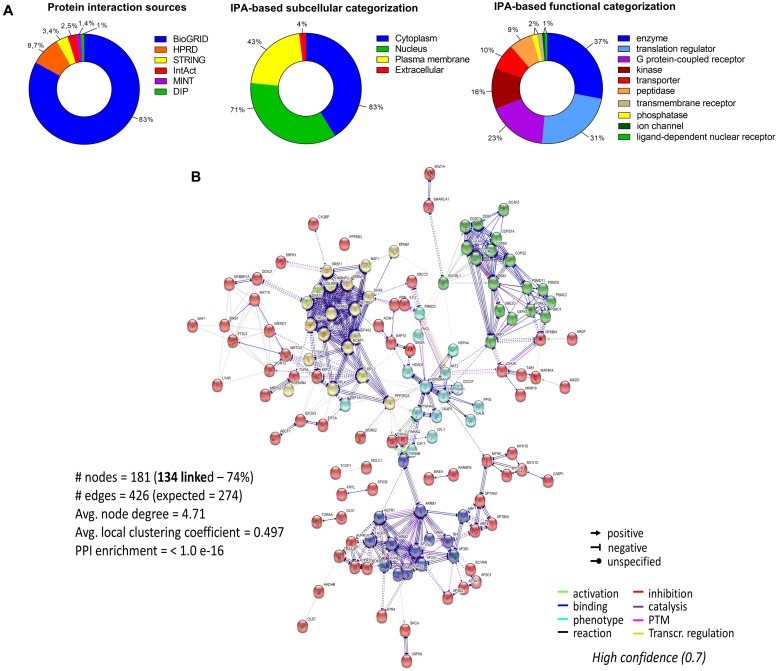
Molecular analysis of the functional GRK5 interactome. The interactors of a protein are indicative of its function. Thus, to further investigate the function of GRK5 we extracted empirically identified GRK5 interacting partners from multiple informatics databases, BioGrid (https://thebiogrid.org/), HPRD (Human Protein Reference Database: http://www.hprd.org/), IntAct (https://www.ebi.ac.uk/intact/), MINT (The Molecular INTeraction Database), STRING (https://string-db.org/) and DIP (Database of Interacting Proteins: http://dip.mbi.ucla.edu/dip/). This curation generated a list of 183 proteins which are proven interactors of GRK5 **(A)**. The left panel indicates the distribution of the curated proteins from the aforementioned databases. This dataset was further analyzed using Ingenuity Pathway Analysis (IPA) to first create an unbiased assessment of the subcellular distribution (center panel) of the interactors and then secondly a functional categorization of the GRK5 interactors (right panel). Using our curated 183 protein input we employed STRING to investigate the strength of interactions between these diverse GRK5 interacting proteins. With this we applied a “strong” cut-off strength of confidence (0.7) and also limited the protein-protein interaction types to those empirically observed in physical interaction of co-expression experiments. In addition, all unconnected nodes were removed from the network **(B)**. K-means clustering was employed to group the interacting proteins in the network into five main clusters (blue, red, yellow, mint, and dark green).

**FIGURE 3:**
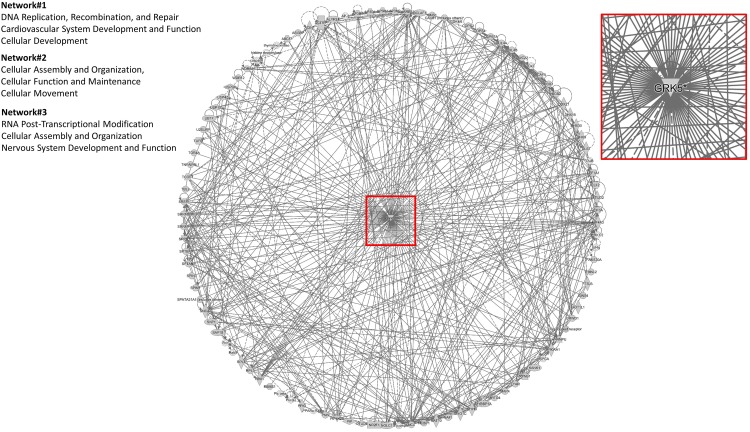
IPA-mediated creation of a radial hierarchical super-network. A radial hierarchical “supernetwork” was constructed using the IPA network construction suite. Through this analysis the three highest scoring networks (based on numbers of interacting proteins from the primary input list of 183 GRK5 interactors) were associated with “*Cellular organization and movement*,” “*Cardiovascular System Development and Function*,” and “*Nervous System Development and Function*.” These networks carry physiologically relevant functions germane to the central hypothesis of this review: GRK5 as a functional bridge between cardiovascular and neurodegenerative disorders. Upon merging these top three scoring protein networks we discovered that they all centrally converged upon GRK5 itself.

**FIGURE 4:**
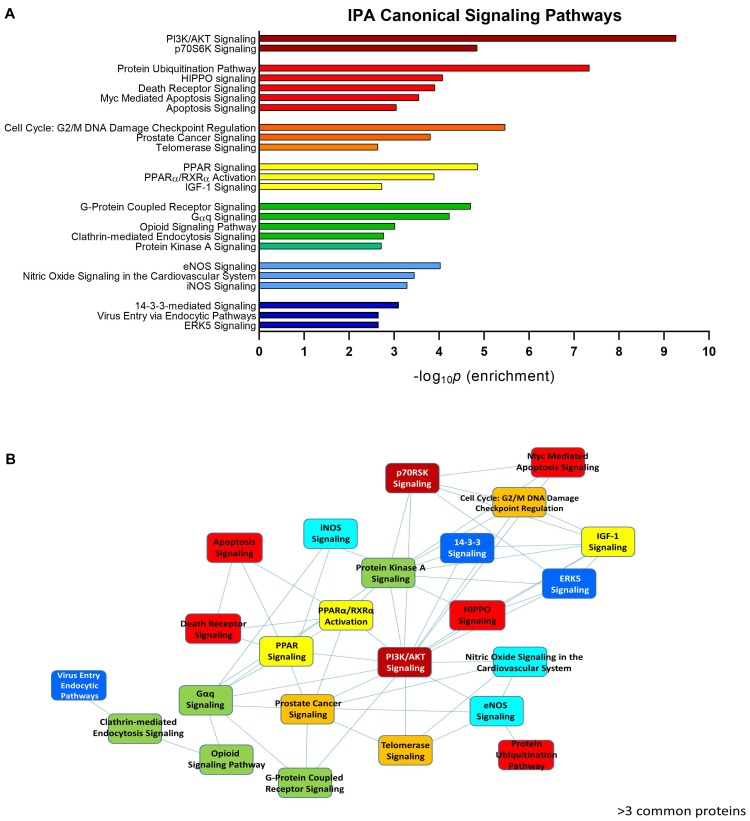
IPA-assisted canonical signaling pathway analysis for the GRK5 interactome. The canonical signaling pathway suite of IPA was employed to generate a signaling cascade appreciation of the functional activity of the GRK5 interactome. **(A)** Indicates the top 20 most significantly (*p* < 0.05) enriched canonical signaling pathways generated with our curated GRK5 interactome. The pathways are color coded and clustered into different functional groups. The close association between these 20 pathways (mediated by common proteins involved in multiple signaling pathways: ≥3 proteins in common) is depicted by the pathway network diagram in **(B)**.

To assess the current state of the metadata concerning the known functional interactome of GRK5 (Figure 2A – left panel), we extracted binding partner identities from BioGrid^1^, HPRD^2^, IntAct^3^, MINT^4^ (The Molecular INTeraction Database), STRING^5^ and DIP (Database of Interacting Proteins)^6^. The cumulated known physical interaction partners for GRK5 are detailed in the annotated Supplementary Table S1. The diversity of subcellular distribution (Figure 2A – center panel) and molecular function (Figure 2A – right panel) of these interactors were categorized using Ingenuity Pathway Analysis (IPA)-based annotation of the extracted GRK5 interactome metadata. The potential functional relationships between the protein factors in the GRK5 interactome were then analyzed with STRING functional network association analysis (Figure 2B). Using a high strength reliability cut-off, a strong functional connectivity between 134 of the 183 known GRK5 interactors was found. This GRK5 associated network demonstrated a very high enrichment probability for the represented network (i.e., *p <* 10e^-16^). This network contained several functional groups (assessed with unbiased k-means clustering) associated with: (*i*) classical GPCR functionality (group 1-Blue); (*i*) stress- and DNA damage-responsiveness (group 2 – Dark Green); (*iii*) protein chaperoning (group 3 – Mint); (iv) RNA metabolism and transcriptional control (group 4 – Yellow). Reinforcing this unbiased appreciation of the known molecular functionalities of the GRK5 interactome we found, using the network-building suite of the IPA platform, that similar functions (i.e., DNA Replication, Recombination, and Repair, RNA Post-Transcriptional Modification) were enriched in the top three most highly enriched signaling networks created using the metadataset of known GRK5 interactors (Figure 3). In addition to these functions reminiscent of the STRING-based predictions, we also noted that physiologically-relevant functions germane to the central hypothesis of this review were also evident related to these networks, i.e., “Cardiovascular System Development and Function” and “Nervous System Development and Function.” Merging the top three highest scoring IPA-generated networks and allowing the unbiased IPA-mediated creation of a radial hierarchical super-network, revealed that indeed with simple database cross-analysis, GRK5 was evidently the central nexus of control of this aggregated dataset. Beside the generation of functional insights from protein–protein interaction networks within the GRK5 interactome, we also investigated this metadata from the aspect of IPA-assisted canonical signaling pathway analysis (Figure 4A). Using our standard cut-offs of signaling pathway enrichment (i.e., >2 proteins per pathway, with an enrichment probability *<*0.05) we found that from a signaling cascade perspective the GRK5 interactome was associated with multiple signaling systems linked to cellular protective activity (e.g., PI3K/AKT Signaling), apoptotic regulation (e.g., Myc Mediated Apoptosis Signaling), DNA damage repair and aging (e.g., Telomerase Signaling), metabolic activity (e.g., PPAR signaling), GPCR regulation and cardiovascular system control (e.g., Nitric Oxide Signaling in the Cardiovascular System). Applying a cut-off of at least three shared proteins between these diverse signaling pathways we found that indeed functional links between all of these cascades were evident (Figure 4B). This close connectivity indeed suggests that through a common association with GRK5, the different proteins populating these signaling systems are likely to coordinate these distinct activities in concert across the multiple tissues where GRK5 is expressed. Therefore, using unbiased informatic analyses of our curated GRK5 interactomic metadata, we have been able to demonstrate multiple insights into GRK5 functional biology and also reinforce our central post that GRK5 can act as an age-related bridge between cardiovascular and neurological pathomechanisms. A more detailed appreciation of the GRK5 interactome and its functional signaling spectrum will likely assist in the derivation of potentially new signal-specific therapeutics that exploit this signaling paradigm in a beneficial manner. In addition, our expanded understanding of GRK5 interactomics also helps place its comprehensive signaling activity in the context of whole-somatic “programs” of related molecular signatures.

### Role(s) of GRK5 in Molecular Aging

GRK activity has been linked with multiple age-associated neoplastic, metabolic, neurodegenerative and cardiovascular ailments (Premont and Gainetdinov, 2007; Gurevich et al., 2012). At the specific disease level, the expressional regulation and activity of GRK5 has been linked with multiple age-related diseases such as type 2 diabetes mellitus (T2DM) (Li et al., 2013), cardiac hypertrophy (Gold et al., 2012), hypertension (Harris et al., 2008), Parkinson’s disease (Arawaka et al., 2006; Bychkov et al., 2008), and Alzheimer’s pathology in mice and humans (Suo et al., 2007). As we age, a progressive dysfunction of multiple receptor signaling systems, across a broad range of tissues, takes place. In this context of age-related receptor system dysfunction, the loss of signaling system sensitivity has been most intensively studies for the insulinotropic signaling cascade. Disruption of the ability to effectively sense, uptake and eventually metabolize glucose has been identified as a pivotal regulator of the rate of aging in nearly every animal model tested (Ma and Gladyshev, 2017). Many of the first genes identified in lower species that control animal longevity were almost exclusively associated with the insulinotropic/insulin-like growth factor system (Mathew et al., 2016). The glycometabolic system, as well as the somatic sensitivity to insulin receptor functionality, is also strongly controlled through the functional status of adipose tissue in the body, e.g., adiponectin release from white adipose tissue is a potent insulin sensitizing factor (Diez and Iglesias, 2003). Commensurate with a potentially important role of GRK5 in aging, it has been shown to be strongly expressed in adipose tissues, suggesting that its functionality may impact the glycoregulatory system. Wang F. et al. (2012) demonstrated that GRK5 genomic deletion in murine models resulted in the generation of significant insulin resistance. In addition to this, genetic polymorphisms of GRK5 have been strongly associated with the generation of T2DM (Xia et al., 2014) and the efficacy profile of anti-diabetic therapeutic agents (Shang et al., 2018). Furthermore, previous studies performed in GRK5 knock-out mice (GRK5-KO) reinforced the importance of GRK5 in metabolism as these animals displayed a decreased white adipose tissue mass, a lower weight gain, a decreased expression of adipogenic genes and a reduced adipocyte differentiation when fed a high-fat diet (Wang F. et al., 2012; Wang L. et al., 2012). Although human data linking GRK5 to metabolism are sparse, a recent genome-wide association study found a robust association of two single nucleotide polymorphisms (SNPs) in the GRK5 gene with apoB levels and total LDL-cholesterol, highlighting the role of GRK5 in cholesterol metabolism.

As well as long-term dysfunction of metabolic signaling systems in the aging process, significant disruptions of inflammatory mediator receptor systems are evident. This inflammatory signaling perturbation typically results in the creation of chronic low-grade inflammatory syndrome, recently codified as “inflammaging” (Baylis et al., 2013; Franceschi et al., 2018; Olivieri et al., 2018). Inflammaging, as a process, has been proposed to be functionally independent of exogenous systemic infection (Franceschi et al., 2000; Frasca and Blomberg, 2016). This chronic inflammatory condition has been linked to potentiated circulatory C-reactive peptide and IL-6 (interleukin 6) concentrations. Protracted exposure to these pro-inflammatory agents predisposes patients to an increased incidence of obesity, premature immunological aging, vascular sclerosis and neurodegenerative phenotypes (Tabas, 2010; Barzilai et al., 2012). The inflammaging process itself appears to be closely tied to the mechanisms of whole-somatic aging trajectory control. Hence, inflammaging has been strongly linked to the potentiation of nuclear factor-κB (NF-κB) activity – a process that at the hypothalamic level seems to act as an arbiter of the aging process (Salminen et al., 2012; Zhang G. et al., 2013).

At a fundamental level NF-κB has been shown possess the ability to regulate the expression of GRK5 (Islam et al., 2013) – therefore these two systems in fact potentiate each other’s activity in a feed-forward loop, a mode of signaling highly characteristic of the aging process itself. Demonstrating the intersection of GRK5 with the inflammatory aging process, GRK5 has been shown to antagonize TLR4 (Toll-like receptor 4) mediated phosphorylation of the NF-κB p105 protein. This action inhibits inflammatory mediator (lipopolysaccharide) sensitivity in macrophages (Parameswaran et al., 2006). Subsequent to the discovery of GRK5 regulation of p105, Sorriento et al. (2008) reported that GRK5 binding to IκBα stabilizes this protein and facilitates the nuclear accumulation of IκBα by masking and thus inhibiting its nuclear export signal sequence. This nuclear accumulation of IκBα can then lead to decreased NF-κB activation in vascular endothelial cells. Research from Wu et al. (2012) employing a GRK5 knockout (KO) murine model confirmed that endothelial GRK5 likely stabilizes IκBα in a manner reminiscent to previous studies (Rockman et al., 1996; Sorriento et al., 2008). Using this model, Islam et al. (2013) further demonstrated an NF-κB inhibitory action of GRK5 in cardiac muscle cells.

Taken together, GRK5 is clearly a vital component in both energy metabolism and chronic inflammation paradigms. It is interesting to note that both of these systems are known to strongly control molecular aging pathologies implicated in many different human disorders and consequently in inflammatory pathways (Packiriswamy et al., 2013). These findings therefore make GRK5 a potentially important therapeutic target in the treatment of age-related diseases such as cardiovascular disease, neurological and metabolic disorders. In this review, we discuss the role of GRK5 in the context of cardiovascular and neurodegenerative disease to emphasize its function in inflammaging.

#### The Role of GRK5 in Cardiovascular Disease Pathology

Cardiovascular pathophysiologies, such as myocardial ischemia, myocardial infarction or hypertension involve the dysregulation of cardiac GPCR responsiveness, which in turn is partly induced by deleterious GRK signaling activity profiles (Dorn, 2009; Cannavo et al., 2013). The first cardiac GRK form identified was GRK2 (Kwatra et al., 1989), while the discovery of cardiac GRK5 came later (Premont et al., 1994). GRK5 was found to be highly expressed in the myocardium through several studies employing genetically engineered mice with altered GRK5 levels (Kunapuli and Benovic, 1993; Premont et al., 1994; Premont and Gainetdinov, 2007). Homozygous GRK5-KO mice are born with a normal basal phenotype, although a loss of both GRK5 and GRK6 in mice results in lethality (Gainetdinov et al., 1999; Burkhalter et al., 2013). Further studies in zebrafish lacking the GRK5 homolog Grk5l, suggested the importance of GRK5 fine tuning capacity in cardiac development through the mTOR pathway. Hence, these Grk5l deficient fish demonstrated altered cardiac tissue generation associated with premature loss of muscle cell progenitors leading to an imbalance of gross structure (Burkhalter et al., 2013; Philipp et al., 2014). Of note, GRK5 is shown to be up-regulated in heart failure (Chen et al., 2001). It has been demonstrated that elevation of GRK5 expression in vascular smooth muscle cells (VSMCs) can also induce the development of high blood pressure (Harris et al., 2008) via altered β1-adrenergic receptor (β1-AR) and angiotensin II (Ang II) receptor signaling dynamics (Eckhart et al., 2002; Keys et al., 2005). GRK5 functionality also appears to be linked to the generation of atherosclerotic vascular pathophysiologies. Hence, the genomic deletion of GRK5 in an ApoE4-deficient murine background significantly accelerated the creation of aortic atherosclerosis compared to control mice (Wu et al., 2012).

##### Cardiac failure

GRK5 appears to exert a pivotal role in cardiac failure and several cardiomyopathies including cardiac hypertrophy (Dzimiri et al., 2004; Gold et al., 2012). Cardiac hypertrophy refers to the abnormal enlargement, or thickening, of cardiac muscle. This thickening can be caused by increases in cardiomyocyte size themselves or via changes in other cardiac muscular components, such as extracellular matrix. Cardiac hypertrophy can be induced via physiological effects (e.g., elevated cardiovascular exercise) or as the result of pathophysiology (e.g., hypertension or valvular disease) (Tardiff, 2006).

In humans, four non-synonymous SNPs of GRK5 with translational significance have been demonstrated. Of these known SNPs, the RH-domain resident Q41L polymorphism [leucine (L) converted to a glutamine (Q)] is highly enriched amongst African-American (A-A) individuals (Liggett et al., 2008). This divergent form of GRK5 possesses an augmented capacity to desensitize β2-adrenergic receptors (β2ARs) (Wang et al., 2008), thus engendering a population specific cardiovascular effect. The Q41L GRK5 variant appears to afford protection against congestive cardiac failure amongst A-A heart failure patients (Eijgelsheim et al., 2008; Liggett et al., 2008). Reinforcing the potential protective capacity of GRK5 in the cardiac setting, increased GRK5 expression has been shown to attenuate cardiac burden in response to intense adrenergic stimulation (Chen et al., 2001; Brinks et al., 2010). As expected, a GRK5 activity blockade mediates the opposite effect, i.e., increased cardiac performance as well as improved resilience in the context of heart failure (Raake et al., 2008; Vinge et al., 2008). It has also been shown that functional GRK5 inhibition, performed by ectopic expression of an N-terminal GRK5 peptide fragment of GRK5, reduces the extent of cardiac muscle damage and attenuates the risk of heart failure (Sorriento et al., 2010).

During cardiac failure, the expression and activity of GRK5 are reflexively increased to enhance β-adrenergic receptor desensitization and thus attenuate contractility (Chen et al., 2001). Activation of GPCRs by hypertrophic agonists, such as phenylephrine and/or Ang II, engages a number of intracellular signaling pathways, including calcineurin-nuclear factor of activated T cells (NFAT) (Molkentin et al., 1998), Ca^2+^/CaM – dependent kinase II (CamK II) (Zhang et al., 2007; Bossuyt et al., 2008), MAPKs (Purcell et al., 2007; Kehat et al., 2011) and the Akt-mechanistic target of rapamycin (mTOR) pathway (Shioi et al., 2002; Sussman et al., 2011) among many others, that are important transducers of the hypertrophic response.

GRK5 can undergo nuclear translocation in a calmodulin-dependent manner following Gα_q_-based signals emanating from α-adrenergic and Ang II receptors. This nuclear translocation of GRK5 has been shown to be mutually exclusive with its interaction with plasma membrane GPCRs – thus distinguishing canonical and non-canonical GRK5 functions (Gold et al., 2012, 2013). This cellular redistribution is proposed to help mitigate the deleterious functions of cardiac hypertrophy (Yi et al., 2002; Johnson et al., 2004, 2013; Martini et al., 2008; Zhang et al., 2011; Gold et al., 2013). Nuclear GRK5 migration is assisted through a productive interaction with calcium sensing proteins (CSP) (Akhter et al., 1998) – thus GRK5 is specifically sensitive to the presence of Ca^2+^/CaM (Freeman et al., 1998; Haeseleer et al., 2000). Indeed, GRK5, possessing a high affinity for CaM, is rapidly inactivated in cells upon elevations in cytosolic calcium. This aspect of GRK5 biology reinforces its pivotal role in the modulation of calcium-associated muscular contractility (Ikura, 1996; Schafer and Heizmann, 1996).

It has been demonstrated that nuclear GRK5 acts as a class II histone deacetylase kinase (HDAC). In this scenario it has been reported that GRK5 is able to phosphorylate HDAC5 (histone deactylase 5) (Johnson et al., 2013). This GRK5-mediated phosphorylation causes redistribution of HDCA5 out of the nucleus resulting in a function alleviation of its MEF2 (myocyte enhancer factor 2) transcription factor repression – leading to “de-repression” of MEF2. Demonstrating the important role of GRK5 in cardiovascular aging this GRK5-mediated MEF2 activation transcribes multiple genes associated with cardiac hypertrophy (Martini et al., 2008; Johnson et al., 2013). GRK5 activity has further been shown to control hypertrophic responses via its interaction in the nucleus with components of the NFAT pathway (Hullmann et al., 2014). GRK5 interacts with the NFAT-pathway in the nucleus during pathological hypertrophy. In addition, it is clear that GRK5 is strongly connected with the NF-κB signaling cascade (Parameswaran et al., 2006; Sorriento et al., 2008; Patial et al., 2009; Valanne et al., 2010; Islam and Koch, 2012; Wu et al., 2012; Islam et al., 2013) as an NF-κB binding element has been identified within the GRK5 DNA promoter region. This functional signaling region has subsequently been demonstrated to orchestrate the expression pattern of GRK5 in cardiac muscle cells (Islam and Koch, 2012).

Physiological hypertrophy does not only occur naturally in the heart due to augmented exercise regimens but also during pregnancy (Dorn, 2007). Non-pathological cardiac hypertrophy is a process typified by relatively normal and proportionate myocyte growth – this reflexive response increases the capacity for cytoprotective cardiac activity (Huang et al., 2011; van Berlo et al., 2013). In contrast, pathological hypertrophy involves a disruption of the proportions of the new myocytes that causes an eventual diminution of heart chamber volume with a concomitant augmentation of septal wall thickness (van Berlo et al., 2013). Recent research has suggested that GRK5 is only a controller of non-pathological hypertrophy (Traynham et al., 2015). In this study, using TgGRK5 mice, it was shown that physical exercise induced a classical physiological cardiac hypertrophy response. With specific respect to the nuclear functionality of GRK5 in cardiac hypertrophy it was shown in this exercise context that minimal nuclear GRK5 activity was found (Traynham et al., 2015). This corresponds with a study demonstrating that NFAT was not shown to regulate physiological hypertrophy (Wilkins et al., 2004). While elevated levels of intracellular Ca^2+^ levels are common to both physiological and pathological cardiac hypertrophy, it has been proposed that pathological hypertrophic effects are differentially controlled through distinct intracellular calcium stores. Thus differential sources of “activating” calcium may allow the specific stimulation of the GRK5-related hallmarks of pathological hypertrophy, i.e., nuclear GRK5 accumulation, HDAC kinase activity and increased NFAT activity. Reinforcing the concept of differential hypertrophic mechanisms, none of these selective events are routinely found in standard exercise-induced hypertrophic paradigms (Traynham et al., 2015).

While GRK5 is evidently associated with deleterious cardiac signaling and cell growth, GRK5 does appear to possess additional non-pathological roles in heart functionality. For example, GRK5 has been shown to be an important intermediate in the mitogenic and pro-survival signaling cascades emanating from the β1-adrenergic receptor-mediated transactivation (Maudsley et al., 2000) of the epidermal growth factor receptor (EGFR) (Noma et al., 2007). Therefore, it appears that GRK5 possesses a dual functionality with respect to cardiac activity, i.e., GRK5 is involved in both protective and detrimental signaling events that are delineated via differential subcellular compartmentalization between nuclear and non-nuclear sites.

##### Hypertension

The maintenance of well-controlled vascular blood pressure is imperative for effective and reliable delivery of oxygenated blood to all major life-preserving organs. Significantly and chronically elevated blood pressure, i.e., hypertension, is a prominent risk-determining player in the etiological profile of multiple chronic conditions including ischemic heart disease with associated subsequent cardiac and renal failure (Bath et al., 2003; Chobanian et al., 2003). The major organs and processes that endogenously regulate vascular pressure include the kidney, heart and the contractile state of VSMCs which regulates radial changes of blood vessels, thus modulating peripheral vascular resistance.

High blood pressure, with its associated stressful effects on vascular wall integrity, can result in the potentiation of GRK5 expression within VSMCs. Associated with these observations it has been shown that Ang II stimulation of VSMCs can also increase GRK5 expression levels in a calcium dependent manner (Ishizaka et al., 1997). This association between VSMC-based GRK5 expression and hypertension was again studied by Keys et al. (2005) in which an ectopic increase of GRK5 expression in vessels was genetically engineered. GRK5 overexpression was subsequently found to induce a gender-specific hypertensive response, i.e., blood pressure increases were much more profound in males compared to females (Keys et al., 2005). Both male and female hypertension in these GRK5-overexpressing mice was ablated upon treatment with the inhibitor of Gα_i_ signaling, pertussis toxin. Further gender-specific effects on the cardiovascular parameters of these GRK5-overexpressing mice were also apparent, e.g., β1-adrenergi receptor signaling in males was altered while Ang II-mediated increased vascular tone was only found in females (Keys et al., 2005). Interestingly, and in contrast to the reported overexpression of GRK2 in VSMCs, the elevation of GRK5 expression failed to induce any significant cardiac hypertrophy (Eckhart et al., 2002).

##### Atherosclerosis

Atherosclerosis presents as a long-term inflammatory disease found primarily in the major arteries. This condition is typified by the accumulation of oxidized low-density lipoproteins (LDL) within the arterial wall and a progressive inflammatory cell infiltration into the vessel (Ross, 1999; Braunersreuther et al., 2007). The recruitment of inflammatory cells to these lesions is triggered by the production of chemokines within the plaque microenvironment (Braunersreuther et al., 2007). Chemokine-stimulated GPCRs initiate several downstream effectors, promoting actin polarization, shape changes and directed cell movement which ultimately leads to atherosclerotic plaque formation (Kehrl, 1998).

GRK5 possesses the capacity to regulate signaling through multiple heptahelical receptors (Pitcher et al., 1998; Premont and Gainetdinov, 2007) including multiple types that have been strongly linked to etiological activities in the atherosclerotic process (Hayek et al., 1999; Tiruppathi et al., 2000; Fan and Malik, 2003; Kim et al., 2005; Bea et al., 2006; Zernecke et al., 2008). Interestingly GRK5 has also been shown to phosphorylate other signal transduction proteins that can influence the atherosclerotic process too, including p53 (Mercer et al., 2005), IκBα (Patial et al., 2009, 2011), platelet derived growth factor receptor-β (PDGFRβ) (Wu et al., 2006; Cai et al., 2008) and HDCA5, via MEF2 activation (Martini et al., 2008). GRK5 can also stimulate anti-atherogenic signaling activity in model systems. For example, GRK5-KO mice have an increase in lesion area when compared to wildtype mice through two different cell-type regulatory mechanisms in monocyte/macrophages and VSMCs (Wu et al., 2012). In VSMCs, GRK5 is able to promote the degradation of the pro-atherogenic platelet-derived growth factor receptor-β in lysosomes which is thought to reduce platelet-derived growth factor-mediated VSMC proliferation and migration (Wu et al., 2012). GRK5 also regulates monocyte chemotaxis; i.e., *in vitro* GRK5-KO monocytes possess increased migration capacity in response to C-C chemokine ligand 2 (CCL2) (a ligand for the C-C chemokine receptor type 2 (CCR2) receptor) and colony stimulating factor-1 (CSF1) (a ligand for the colony stimulating factor 1 receptor (CSF1R) tyrosine kinase) (Wu et al., 2012). CCL2-mediated leukocyte migration is instrumental in atherosclerotic lesion progression and responsible for the increased macrophage content in lesions from GRK5-KO mice. These findings highlight the potential mechanisms in both monocyte retention and emigration after their migration across the endothelium and present new strategies to limit atherosclerotic lesion progression.

#### GRK5 in Neurodegeneration

In contrast to its expression profile in cardiovascular organs, central nervous system (CNS) expression of GRK5 is comparatively sparse (Kunapuli and Benovic, 1993; Premont et al., 1994) because of a low GRK5 expression in the majority of cortical areas, except for the limbic system (Erdtmann-Vourliotis et al., 2001). As we have outlined previously there is emerging evidence that demonstrates the multiple non-canonical roles of GRK5 outside of GPCR activity regulation. These novel effects of GRK5 are also associated with multiple important neurophysiological functions. For example, GRK5 deficient mice display a specific and nuanced subtype-specific muscarinic receptor dysfunction while closely-associated adrenergic and opioid receptor activity was not altered (Gainetdinov et al., 1999; Matsui et al., 2004; Liu et al., 2009). CNS muscarinic receptor activity has long been associated with the maintenance of learning and memory behavior (Blokland, 1995). Thus, it is unsurprising that GRK5-KO mice present with cognitive dysfunction shown to correlate with hippocampal neurosynaptic failure (Liu et al., 2009). Again, as with the cardiovascular effects, gender differences in GRK5 activity were seen with respect to neurodegenerative phenotypes, i.e., augmented axonal defects and synaptic degenerative changes, were shown to be greater in female experimental animals as opposed to males. In addition, at the molecular signaling level, hippocampal levels of the synaptosomal-associated protein 25 (SNAP25) and synaptophysin were significantly lower in females compared to males (Liu et al., 2009).

It has also been proposed that the involvement of GRK5 in dementia-related conditions is likely associated with its potent role in regulating neurite outgrowth that is required for optimal learning and memory function (Chen et al., 2011).

Obstructive sleep apnea (OSA) occurs in approximately 2 to 4% of middle-aged women and men, respectively. Among these, OSA is also observed to be more common in obese patients, potentially due to increased tracheal occlusion caused by excessive cervical adipose deposits. While OSA can induce health concerns with respect to lack of effective sleep patterns, it is evident that OSA is also closely associated with intermittent cerebral hypoxia. Considering this deleterious hemodynamic effect it is unsurprising that OSA has been shown to be a potent risk factor for associated cognitive impairment in nearly a quarter of diagnosed OSA patients (Singh et al., 2016). At the molecular level CNS hypoxic episodes can often result in the increased production rate of ROS – these oxygen species can rapidly interact and modify a broad range of CNS lipids, nucleic acids and proteins. Enhanced CNS ROS production has therefore been associated vascular endothelial dysfunction, perturbations of blood-brain barrier integrity and eventual neurosynaptic signal transduction dysfunction. Rodent models of intermittent hypoxia have been developed to effectively replicate the OSA found in human patients (Badran et al., 2014; Singh et al., 2016). Using these, it has been demonstrated that intermittent hypoxia effects upon behavioral rodent activity (anxiety, balance, short-term memory) are acutely sensitive to, and potently augmented by, the genetic deletion of GRK5 (Badran et al., 2014; Singh et al., 2016). Such research suggests that part of the CNS functionality of GRK5 may be associated with oxygen sensation neurochemistry, potentially via controlling astrocytic functions.

##### GRK5 and Alzheimer’s disease (AD) pathology

For a significant period of time, undue focus on amyloid pathologies and their subsequent association with Alzheimer’s disease (AD) has been in effect (Tanzi and Bertram, 2005; Jack et al., 2010; Mawuenyega et al., 2010; Lesne et al., 2013; Selkoe and Hardy, 2016). However, and from a more therapeutically important aspect, there has long been known to be an extant cholinergic receptor (post-synaptic nicotinic and M1 muscarinic acetylcholine) hypofunction evident in AD (Terry and Buccafusco (2003)). In AD it has been demonstrated that augmented presynaptic cholinergic activity results in the reflexive attenuation of synaptic acetylcholine release. This reduced release therefore results in diminished level of activity at the post-synaptic muscarinic M1 GPCRs. Indicating the importance of muscarinic signaling in AD pathophysiology, muscarinic M1 receptor signaling cascades can inhibit β-amyloidogenic (Aβ) amyloid precursor protein (APP) processing, resulting in a decreased level of cytotoxic β-amyloid accumulation (Sadot et al., 1996). From genetic deletion mouse models (i.e., GRK5-KO) it has been shown that GRK5 functionality is associated with severe hippocampal dysfunction (loss of neurosynaptic proteins and axonal swelling) as well as increased amyloidosis (Suo et al., 2007; Li et al., 2009).

When combined with murine AD models (Tg2576) GRK5 deficiency was found to cause increased inflammatory astrogliosis in both hippocampal and cortical brain areas (Li et al., 2008). In addition to this effect, the GRK5 deficiency was also linked with both increased soluble Aβ levels as well as increased insoluble Aβ plaque load (Cheng et al., 2010). These findings were proposed to be due to a GRK5-induced potentiation of presynaptic muscarinic M2 receptor activity that resulted in a significant reduction of synaptic acetylcholine transmission levels (Liu et al., 2009; Cheng et al., 2010). This GRK5-associated alteration of synaptic receptor activity in murine models of AD has been shown to be linked to disruptions in sub-cellular compartmentalization of GRK5. Hence, Zhang et al. (2014) were able to demonstrate that aged AD model mice possess a highly specific plasma membrane deficiency of GRK5 (Zhang et al., 2014). A paucity of pre-synaptic GRK5, with its concomitant detrimental effect on M2-acetylcholine receptor-controlled acetylcholine release, has been subsequently linked to an exacerbation of tau hyperphosphorylation and further neuronal dysfunction. Using chemical blockade of these hyperactivated M2 receptors Zhang et al. (2014) were able to attenuate this tau hyperphosphorylation in a GSK3β-dependent manner.

It is thus apparent that GRK5 may indeed hold the key to the connection between the current major theories of AD, i.e., the amyloid and the cholinergic hypotheses. The cholinergic hypothesis suggests that cholinergic CNS dysfunction is responsible for the cognitive decline (Bartus et al., 1982) while the amyloid hypothesis proposes that Aβ is the AD-causative factor (Bartus et al., 1985; Woolf, 1996; Hardy and Selkoe, 2002; Small and Cappai, 2006; Fisher, 2008). Interestingly, as we have previously outlined, Aβ is thought to be one of the driving forces for alterations of membrane associated GRK5 in AD (Suo et al., 2004). GRK5 plasma membrane deficiencies can mediate presynaptic M2 acetylcholine autoreceptor hyperactivation that, in turn, causes post-synaptic cholinergic hypoactivity through the functional attenuation of cholinergic neurotransmission. This disrupted cholinergic transmission then serves to augment Aβ amyloid production leading to a “feed-forward” process of progressive neurosynaptic dysfunction and amyloid toxicity. In this recursive process both amyloid deposition and cholinergic dysfunction each can serve as a cause and/or consequence of each other, with the extant GRK5 dysfunction as the pivotal mediator. Given the present interest in these hypotheses in AD pharmacotherapy, the importance of GRK5 as a drug target in this system may increase significantly in the future.

As a prelude to our next section, it is intriguing to note that GRK5 can be further connected with AD through its ability to phosphorylate α-synuclein (SNCA) (Pronin et al., 2000; Arawaka et al., 2006; Bychkov et al., 2008), tubulin as well as the AD-associated tau protein (Zhang et al., 2014). This pathological effect has been proposed to occur through GRK5-mediated phosphorylation causing increased SNCA polymerization and eventual aggregation – in a similar manner to that seen with Aβ in the context of AD (Carman et al., 1998).

##### Parkinson’s disease (PD)

Parkinson’s disease (PD) is one of the most commonly encountered neurodegenerative diseases at the present time, just behind AD with respect to world prevalence. The pathological effects of PD impact the primary fine motor systems of the body. PD is clinically typified by progressive deterioration of tremor, rigidity, bradykinesia/akinesia, gait disturbance, and postural instability. The major defining neuropathological feature of PD has long been considered to be the loss of neurons in the *substantia nigra* that provide dopaminergic innervation to the striatum, the CNS region most heavily implicated in fine motor control. Since the molecular mechanism causing dopaminergic neuron dysfunction are yet to be comprehensively defined, there are unfortunately no effective current pharmacotherapeutic interventions capable of retarding, or reversing, the disease (Dawson and Dawson, 2002). One of the lesser known aspects of PD is the fact that advancing age is arguably the strongest risk factor for its generation (Rango and Bresolin, 2018). In this light it is unsurprising that PD is typically is presented after the age of 60.

With respect to the functional intersection between GRK5 and PD pathology, it has been demonstrated by multiple research groups that GRK5 represents one of the major kinases that can phosphorylate SNCA. This classical function of GRK5 results in the promotion of the oligomerization of PD (with actual co-localization of GRK5 and SNCA), facilitating the creation of pathological Lewy bodies in the *substantia nigra* and *locus coeruleus* of PD patients (Pronin et al., 2000; Arawaka et al., 2006; Bychkov et al., 2008). The nuclear functionality of GRK5 is one of its defining functional features among GRK proteins – GRK5 activity itself has also been shown to promote the nuclear translocation of SNCA and its associated factors PLK2 and 3 (Polo-like kinase 2 and 3) (Goncalves and Outeiro, 2013; Fares et al., 2014). While the full ramifications of nuclear SNCA remain currently cryptic, it has been proposed that this aspect of SNCA biology may be independent of the classically-pathological SNCA aggregation modality. It is important to note, especially with respect to aging pathomechanisms, that oxidative stress environments promote the enhanced nuclear localization of SNCA (Xu et al., 2006; Monti et al., 2010; Siddiqui et al., 2012). Within the nuclear domain SNCA has been shown to functionally antagonize histone acetylation, resulting in increased neurotoxicity (Goers et al., 2003; Kontopoulos et al., 2006). Nuclear SNCA has also been found to be a transcriptional regulator capable of binding to PGC1-α (Peroxisome proliferator activated receptor gamma coactivator 1-alpha) promoter regions, and in doing so, potentially regulate mitochondrial gene transcription and thus neurometabolic ROS-associated activity (Siddiqui et al., 2012). In addition to these cell signaling-based analyses, genetic association studies have proposed a haplotypic association of GRK5 gene with the clinical presentation of sporadic PD. These pathological haplotypes associated with functional GRK5 SNPs that can control multiple transcription factors (Yin Yang-1 (YY1) and cAMP response element-binding protein (CREB-1)) that together are capable of potentiating SNCA transcription (Arawaka et al., 2006). Unfortunately, and as is quite common with genetic association studies, subsequent studies have failed to reproduce some of these propositions. Hence, studies employing GRK5 deletion in cells have failed to find a resultant attenuation of SNCA phosphorylation (Sakamoto et al., 2009; Liu et al., 2010). In addition, further studies have not observed a strong localization of GRK5 in Lewy bodies (Takahashi et al., 2006) or a firm association of GRK5 SNPs with PD (Tarantino et al., 2011).

### Meta-Analysis of Diverse Molecular GRK5 Interactors

In our previous section (Molecular Functionality of GRK5) we applied multiple unbiased informatic pipelines to our extracted GRK5 interactome metadata to demonstrate that these diverse proteins do indeed possess concerted and interconnected molecular functions. Using a latent semantic analysis platform (GeneIndexer) (Cashion et al., 2013; Chen et al., 2013; Maudsley et al., 2018) we were able to prioritize multiple, functionally diverse GRK5 interactome factors that possessed the strongest textual associations with input interrogator terms describing, aging of cardiovascular and nervous systems. These proteins included L-3-hydroxyacylcoenzyme A dehydrogenase type II (HADH), 5-hydroxytryptamine receptor 4 (HTR4), GPCR-kinase interacting protein-1 (GIT1), histone deacetylase 6 (HDAC6), and eukaryotic elongation factor 2 (EEF-2). In the following sections we shall detail how these functionally diverse proteins, informatically prioritized from our unbiased GRK5 interactome metadata, still generate a dimensionally-condensed signature of the greater role of GRK5 in somatic coordination of cardiovascular and neurological deterioration with aging.

#### Enzyme: HADH – Mitochondrial Trifunctional Enzyme Subunit β

L-3-hydroxyacyl-coenzyme A dehydrogenase type II (HADH) acts as an endoplasmic reticulum (ER) amyloid β-peptide-binding protein (ERAB). It has been proposed that HADH can facilitate amyloid-induced neurodegeneration by enhancing Aβ toxicity and accumulation in neurons of AD patients. Investigations by Frackowiak et al. (2001) showed the absence of HADH in amyloid plaques or vascular amyloid, but did however denote the expression of HADH in VSMCs in both juvenile and aged control subjects as well as in amyloid free blood vessels in AD cases (Frackowiak et al., 2001). In this respect a potential interaction between HADH and Aβ in amyloid-producing cells was further studied in isolated VSMCs from CNS vessels presenting with an Aβ-related angiopathic condition. HADH had a mitochondrial localization (Zhou et al., 2012; Ibdah et al., 2001) and failed to co-localize with classical endoplasmic reticulum marker proteins. Aβ accumulating cells were those with a low HADH expression. However, the association between low HADH expression levels and Aβ depositions by brain VSMCs requires further studies (Frackowiak et al., 2001).

#### GPCR: HTR4 – 5-Hydroxytryptamine Receptor 4

The 5-hydroxytryptamine receptor type 4 (HTR4) appears to be the predominant cognate receptor responsible for serotonin responsivity within cardioventricular tissue in experimental models of congestive heart failure. HTR4 receptor expression contributes to positive inotropic responses and the productive signaling activity of these receptors is increased upon pathological transitions to heart failure. The HTR4 mediates positive inotropic responses to LV dilatation, as seen in post-infarction congestive heart failure (Brattelid et al., 2007). HTR4 mRNA levels are increased in male Wistar rats with increasing left ventricular hypertrophy and elevated further aging with increasing left ventricular (LV) hypertrophic failure. Therefore, the HTR4 can be differentially induced in LV hypertrophy and failure.

Experimental studies have also demonstrated that the HTR4 serotonin receptor is a prime controller of cognitive activity, depressive conditions and also the etiology of AD. Positron-emission tomographic studies of HTR4 CNS expression patterns across lifespan, using the selective ([11C]SB207145 ligand), found a gender-specific variation in expression profiles, i.e., HTR4 levels showed a profound decline in limbic system areas only in female subjects (Madsen et al., 2011). The deficits of HTR4 receptor expression found in women suggests a role for HTR4 receptors in cognitive and emotional control and may eventually contribute to the higher rate of affective diseases coincident with AD in female patients (Madsen et al., 2011).

#### Kinase: GPCR-Kinase Interacting Protein-1 (GIT1)

The GPCR-kinase interacting protein (GIT) family of proteins (GIT1 and GIT2) were originally identified as GRK and GPCR interacting proteins (Premont et al., 1998). GIT1 is a multifunctional scaffold protein that possesses an ADP-ribosylation factor GTPase activating capacity. With respect to the intersection between GIT1 activity and cardiovascular functionality it has been demonstrated using GIT1 knockout (GIT1-KO) mice that loss of this receptor-associated protein caused structural and functional changes in cardiac mitochondria (Pang et al., 2011). In addition, this group found that several mitochondrial regulator genes (PGC-1α, PGC-1β, Tfam) were also profoundly reduced in the hearts of GIT1-KO mice. As expected, these mice subsequently present with reduced ATP-synthetic capacity and a strong increase in cardiac muscle apoptosis (Pang et al., 2013).

GIT1 genomic deficiency also has been shown to profoundly attenuate vascular smooth muscle growth capacity (Pang et al., 2013). Using a specific GIT1 deletion model of aortic smooth muscle cells Cyclin D1, a key cell cycle regulator, was found to be strongly downregulated significantly decreased in GIT1 knockout cells. Pang et al. (2013) continued to demonstrate that GIT1-associated muscle proliferation control occurred in a PLC-γ- and ERK1/2-sensitive manner. Further linking GIT1 functionality to vascular control in an aging paradigm, GIT1 has been shown to be a novel eNOS (endothelial nitric oxide synthase) binding partner. The association of GIT1 with eNOS has been shown to enhance the catalytic activity of this synthase and therefore suggests that GIT1 is an important controller of vascular relaxatory behavior. Interestingly, genomic ablation of GIT1 results in the opposite functional effect upon nitric oxide synthesis. GIT1 expression has also been shown to be reduced in vascular endothelial cells following hepatic damage (Shikata et al., 2003; Zhang et al., 2005; Jones et al., 2009; Pang et al., 2009). In this specific scenario, recovery of the endothelial expression of GIT1 was found to reverse the evident endothelial dysfunction found in hepatic damage cases. Re-expression of GIT1 after liver injury rescued the endothelial phenotype. Hence the GRK5-interacting protein GIT1, appears important for eNOS function and thus such an interaction will likely have tremendous import upon vascular disorders involving dysregulated eNOS such as arterial stiffness (Liu et al., 2012; Avolio, 2013).

In addition to its role in regulating nitric oxide synthesis, GIT1 has been linked to muscle cell proliferation as it can exert potent cardiovascular signaling effects via the control of Ang II-induced angiotensin receptor signaling. In a case of elegant research Pang et al. (2008) were able to demonstrate that Ang II-mediated HDAC5 phosphorylation (implicated in the c-Src-PLCγ-CamK II-HDAC5 signaling cascade that controls VSMC gene transcription) was GIT1-dependent (Pang et al., 2008). Within this paradigm the direct interaction of GIT1 and CamK II was required for effective Ang II-mediated HDAC5 phosphorylation. Finally, Pang et al. (2008) found that GIT1 genetic deletion reduced the transcriptional activity of MEF2 induced by Ang II. As GIT1 was selected as a binding partner of GRK5 in the meta-analysis, these findings reinforce the pivotal role of the non-canonical activity of GRK5 in the cardiovascular system which is largely associated with its nuclear HDAC5 kinase activity.

#### Transcriptional Regulator: HDAC6

As a cluster of enzymes, the primary role of histone deacetylases (HDACs) is to remove acetyl groups from an N-acetyl lysine amino acid on histone proteins. This histone deacetylation allows for a more compact and efficient packing of DNA with these histones. HDACs, as a protein family, are proteins grouped into four functional classes (I, II, III, IV). Class I, II, and IV possess a zinc dependent active site – these so-called “classic” HDACs are also typified by their enzymatic sensitivity to inhibition by trichostatin A. Class III HDACs comprise a family of trichostatin-insensitive, NAD+-dependent, proteins that are also referred to as “sirtuins” (de Ruijter et al., 2003; Vaquero et al., 2007). With specific respect to the GRK5-interacting HDA6, this protein is classified as a Class IIb HDAC. Demonstrating its close functional association with GRK5 signaling paradigms, HDAC6 expression and activity has been shown to be significantly elevated in stressed cardiac muscle (Lemon et al., 2011), while remaining unchanged in physiological cardiac hypertrophy models. In addition to these *in vivo* analyses, HDAC6 catalytic activity can also be induced by stressful stimuli impinging upon cultured cardiac muscle cells and fibroblasts (Lemon et al., 2011).

In a manner reminiscent to cardiac tissue, HDAC6 levels were found to be potently elevated in CNS regions important for the disease etiology of AD. At the present time research into the role of HDAC6 in AD, it has been proposed that the evident increased HDAC6 expression could be a driving factor in AD-associated neurodegeneration (Zhang L. et al., 2013). While HDAC6 activity has been proposed to be a pro-degenerative factor in the CNS, alternative evidence also points to some potentially neuroprotective functions. For example, molecular targeting of HDAC activity has been shown to be able to directly protect neurons and glia and thus improve physiological outcomes in CNS injury and disease models (Rivieccio et al., 2009).

#### Translational Regulator – EEF2

Evidence has been generated that directly implicates the transcriptional regulator eukaryotic elongation factor 2 (EEF2) in regulating the functionality of cells in response to myocardial ischemia (Demeulder et al., 2013). EEF2 interaction with mTOR and p70S6K appears to generate a regulatory complex that control protein synthesis in times of cellular stress and metabolic aging. This regulatory activity of EEF2 was subsequently shown to be sensitive to the expression levels of AMPKα2. The AMPKα2 protein was demonstrated in this cardiac ischemic models to control p70S6K and EEF2 in normoxic conditions specifically (Demeulder et al., 2013). Besides the role of EEF2 in the cardiovascular system, this elongation factor is found to be active in neurodegenerative signaling paradigms as well. Arguelles and co-workers investigated the role of EEF2 in the hypothalamic-hypophysis system. In old rats it is observed that during aging a considerable diminution of protein synthesis takes place in several tissues, potentially linked to modifications in EEF2. More specifically, research indicated that oxidative stress could be involved in EEF2 post-translational modification, with the resultant formation of covalent malondialdehyde (MDA) and 4-hydroxynonenal (HNE) EEF2 adducts. These long-lasting alterations in EEF2 structure have therefore been proposed to effect the age-dependent attenuation of EEF2-controlled protein synthesis and thus dysfunctional hypothalamic control of neurometabolic activity (Arguelles et al., 2011).

## Discussion

GPCR signaling is an adaptable and highly dynamic process that forms a major component of the current pharmacopeia. Effective control of GPCR signaling dynamics is strongly dependent on several key proteins that regulate signaling sensitivity and post-activation functional fate (Takeda et al., 2002; Cabrera-Vera et al., 2003; Preininger and Hamm, 2004; Oldham and Hamm, 2008; Ritter and Hall, 2009; Hewavitharana and Wedegaertner, 2012). One such family that is intricately linked to the regulation of the activated receptors are the GRKs (Pitcher et al., 1998; Penela et al., 2003; Lefkowitz and Shenoy, 2005). GRKs demonstrate a broad range of activities within multiple physiologically important processes. Commensurate with this importance, perturbations of GRK systems have been linked to diverse pathologies such as bipolar disease (Barrett et al., 2007), AD (Obrenovich et al., 2009), rheumatoid arthritis (Lombardi et al., 1999), multiple sclerosis (Vroon et al., 2005), and PD (Bychkov et al., 2008). This review has focused on one such GRKs, i.e., GRK5 and in particular, the capacity of GRK5 to mediate a signaling connection between cardiovascular and neurodegenerative disease. GRK5 is one of the main cardiac GRK isoforms which is strongly expressed not only in cardiovascular but also CNS tissues. Moreover, unique non-receptor-dependent regulatory roles of GRK5 have been recently uncovered that may prove important for future therapeutic targeting (Kunapuli and Benovic, 1993; Premont et al., 1994; Premont and Gainetdinov, 2007; Schumacher-Bass et al., 2012). Human studies have suggested that GRK5 is increased in expression and activity in various cardiac diseases (Ishizaka et al., 1997; Tardiff, 2006; Gold et al., 2012; Wu et al., 2012) and GRK5 has recently been designated as a potential therapeutic target in cancer due to its anti-tumor effect when inhibited. Molecular inhibition strategies targeting GRKs have been shown to improve cardiac function in several animal models of cardiomyopathy (Raake et al., 2008; Rengo et al., 2009; Volkers et al., 2011; Homan et al., 2014; Gambardella et al., 2016).

To date, one GRK5 inhibitor, amlexanox, has been reported. This agent directly binds the GRK5 kinase domain and hereby inhibits the MEF2 transcriptional domain significantly (Homan et al., 2014). However, amlexanox is not GRK5 selective and yet has to be tested in cancer-related paradigms. To this end, the synthesis of a GRK5 selective inhibitor would be an effective anti-tumor treatment by promoting apoptosis and cell cycle arrest, especially in tumors with a low pro-apoptotic protein p53 abundancy. On the other hand, stimulation of GRK5 expression would be more effective in GPCR-dependent tumors (Gambardella et al., 2016). Xia et al. (2009) has previously performed work in which they classified 1800 compounds that can stimulate CREB activity, a transcription factor of, among other things, the GRK5 gene (Xia et al., 2009) – hence refining molecular CREB regulators may lead to the development of future GRK5 expression regulators.

Alterations of GRK levels, due to both canonical effects on GPCR sensitivity as well as through non-GPCR effects can lead to changes in signaling pathways that regulate apoptosis (Chen et al., 2010), inflammation (Sorriento et al., 2008; Patial et al., 2010) and hypertrophy (Dzimiri et al., 2004; Gold et al., 2012). While canonical cardiac GRK5 signaling can exacerbate the progression to heart failure, novel, non-canonical nuclear GRK5 molecular mechanisms (Martini et al., 2008; Zhang et al., 2011) suggest tremendous future opportunities for pharmacotherapeutic development (Schumacher and Koch, 2017). With the elucidation of novel therapeutically-tractable GPCR biased signaling mediated via β-arrestins, the importance of GRK signaling has received renewed interest. Hence, it has been shown that the specific coterie of GRKs that phosphorylate a receptor can exert a strong functional effect upon the subsequent nature of both G protein-dependent and β -arrestin-dependent signaling functions (Noma et al., 2007; Nobles et al., 2011; Choi et al., 2018). Thus, GPCR phosphorylation by GRKs is a pivotal cellular event as it desensitizes the active GPCR, but also dictates downstream signaling, functionally selecting for downstream G protein pathways or enabling β-arrestin-mediated pathways.

At the present time, it is widely accepted that complex age-related disorders such as cardiovascular and CNS ailments represent highly interconnected molecular events spanning multiple tissues. Moreover, considerable *in vivo* and *in vitro* research in both humans and animals suggests the functional connectivity of cardiovascular disorders with neurodegeneration. To reinforce this plethora of scientific literature, our review particularly focused on the potential function of GRK5 to both physically and functionally bridge cardiovascular and neurodegenerative disorders. Nowadays, a broad range of GRK5 binding partners, connecting many signaling proteins associated with these disorders, have been identified via multiple molecular biological approaches. The extent of such protein–protein interactions can significantly expand the potential of multidimensional molecules to control both health and disease beyond their canonical activities. More specifically, the interaction of GRK5 with non-GPCR proteins has been shown to profoundly influence transduction pathways controlling both CNS and cardiovascular disease trajectories. Hence, here we have assessed the current state of both literature-based data, as well as functional metadata, concerning the known scientific literature and the GRK5 interactome to better elucidate novel mechanisms of intrinsic protein regulation as well to further clarify GRK5-associated physiological signaling proteins. Our unbiased informatic analysis review of the curated GRK5 interactomic metadata, obtained from previously published material, reveals multiple insights into GRK5 functional biology and thus reinforces our central post that GRK5 can act as an age-related bridge between cardiovascular and neurological pathomechanisms. Likewise, physiologically-relevant functions are noted, when analyzing potential functional relationships in the GRK5 interactome, that are germane to the central hypothesis of this review, i.e., “Cardiovascular System Development and Function” and “Nervous System Development and Function.” An additional radial hierarchical super-network revealed GRK5 was as the central controlling nexus of this aggregated dataset. Moreover, we were able to prioritize multiple, functionally diverse GRK5 interactome factors via a latent semantic indexing platform that possessed the strongest textual associations with input interrogator terms describing, aging of cardiovascular and nervous systems. These proteins included HADH, HTR4, GIT1, HDAC6, and EEF2 which generate a dimensionally-condensed signature of the greater role of GRK5 in somatic coordination of cardiovascular and neurological deterioration with aging. A more nuanced appreciation of the GRK5 interactome and its functional signaling spectrum will likely assist in the derivation of potentially new signal-specific therapeutics in the future that exploit this signaling paradigm in a beneficial manner. In addition, our expanded understanding of GRK5 interactomics also helps place its comprehensive signaling activity in the context of whole-somatic “programs” of related molecular signatures. However, the complete ramifications of the correlation between GRK5 levels to different cardiac and neurodegenerative disease etiologies, across the human lifespan, still remains to be determined. Continued investigation will likely reinforce the importance of GRK5 as a therapeutically-exploitable systems-level controller and coordinator of the cardiovascular-dementia pathological axis.

## Author Contributions

JH, JvG, HL, PS-O, RP, BM, and SM all contributed to the writing and editing of this review. JH and SM generated the associated Figure and Supplementary Table S1.

## Conflict of Interest Statement

The authors declare that the research was conducted in the absence of any commercial or financial relationships that could be construed as a potential conflict of interest.

## Footnotes

1 https://thebiogrid.org/

2 http://www.hprd.org/

3 https://www.ebi.ac.uk/intact/

4 https://mint.bio.uniroma2.it/

5 https://string-db.org/

6 http://dip.mbi.ucla.edu/dip/
